# A phase I dose escalation, dose expansion and pharmacokinetic trial of gemcitabine and alisertib in advanced solid tumors and pancreatic cancer

**DOI:** 10.1007/s00280-022-04457-9

**Published:** 2022-07-30

**Authors:** Justin A. Chen, Jasmine C. Huynh, Chun-Yi Wu, Ai-Ming Yu, Karen Matsukuma, Thomas J. Semrad, David R. Gandara, Tianhong Li, Jonathan W. Riess, Kit Tam, Philip C. Mack, Anthony Martinez, Nichole Mahaffey, Karen L. Kelly, Edward J. Kim

**Affiliations:** 1 Division of Hematology and Oncology, Davis Comprehensive Cancer Center, University of California, 4501 X Street, Suite 3016, Sacramento, CA 95817 USA; 2 Bioanalysis and Pharmacokinetics Core Facility, University of California, Sacramento, CA 95817 USA; 3 Department of Biochemistry and Molecular Medicine, University of California, Sacramento, CA 95817 USA; 4 Department of Pathology and Laboratory Medicine, University of California, Sacramento, CA 95817 USA; 5 Gene Upshaw Memorial Tahoe Forest Cancer Center, Truckee, CA 96161 USA; 6 Division of Hematology and Medical Oncology, Icahn School of Medicine at Mount Sinai, New York, NY 10029 USA; 7 Office of Clinical Research, Davis Comprehensive Cancer Center, University of California, Sacramento, CA 95817 USA

**Keywords:** Alisertib, Gemcitabine, Pharmacokinetics, Aurora kinase a, Phase I

## Abstract

**Purpose:**

Aurora Kinase A (AKA) inhibition with gemcitabine represents a potentially synergistic cancer treatment strategy via mitotic catastrophe. The feasibility, safety, and preliminary efficacy of alisertib (MLN8237), an oral AKA inhibitor, with gemcitabine was evaluated in this open-label phase I trial with dose escalation and expansion.

**Methods:**

Key inclusion criteria included advanced solid tumor with any number of prior chemotherapy regimens in the dose escalation phase, and advanced pancreatic adenocarcinoma with up to two prior chemotherapy regimens. Four dose levels (DLs 1–4) of alisertib (20, 30, 40, or 50 mg) were evaluated in 3 + 3 design with gemcitabine 1000 mg/m^2^ on days 1, 8, and 15 in 28-day cycles.

**Results:**

In total, 21 subjects were treated in dose escalation and 5 subjects were treated in dose expansion at DL4. Dose-limiting toxicities were observed in 1 of 6 subjects each in DL3 and DL4. All subjects experienced treatment-related adverse events. Grade ≥ 3 treatment-related adverse events were observed in 73% of subjects, with neutropenia observed in 54%. Out of 22 subjects evaluable for response, 2 subjects (9%) had partial response and 14 subjects (64%) had stable disease. Median PFS was 4.1 months (95% CI 2.1–4.5). No significant changes in pharmacokinetic parameters for gemcitabine or its metabolite dFdU were observed with alisertib co-administration.

**Conclusions:**

This trial established the recommended phase 2 dose of alisertib 50 mg to be combined with gemcitabine. Gemcitabine and alisertib are a feasible strategy with potential for disease control in multiple heavily pre-treated tumors, though gastrointestinal and hematologic toxicity was apparent.

**Supplementary Information:**

The online version contains supplementary material available at 10.1007/s00280-022-04457-9.

## Introduction

Taxanes stabilize microtubules to disrupt the dynamic polymerization and depolymerization necessary for mitosis. However, alteration of normal microtubule dynamics can cause side effects such as neuropathy which is a dose-limiting toxicity of taxanes. Newer generations of mitotic inhibitors are being developed to target proteins present only in cells undergoing active mitosis, thus limiting off-target effects noted with taxanes such as neuropathy.

Aurora Kinase A (AKA) is a member of the Aurora Kinase family of serine/threonine protein kinases. AKA is a serine/threonine kinase highly expressed during G2 transition to mitosis that supports assembly of spindle microtubules and facilitates centrosome maturation [1, 2]. Overexpression of AKA can lead to chromosomal instability [3–5] and has been observed in multiple solid cancers [4, 6–8]. Inhibition of AKA in pancreatic cancer cells causes increased mitotic arrest and apoptosis, leading to decreased proliferation and tumorigenicity [9], with similar apoptotic synergy observed when added to EGFR inhibition in resistant pre-clinical models of *EGFR*-driven NSCLC [8].

Alisertib (MLN-8237) is a potent, highly selective small-molecule inhibitor of targeting the ATP-binding site of AKA with > 200-fold selectivity for AKA compared to Aurora Kinase B [10, 11]. Alisertib, which has yet to be approved for any indication and remains an investigational agent, has demonstrated modest single-agent activity in phase I trials for solid tumors [12, 13]. In vitro and in vivo solid tumor models have suggested enhanced anti-tumor activity with chemotherapy combinations [14–16], and alisertib plus paclitaxel has shown a trend for improved efficacy compared to paclitaxel alone in patients with small cell lung cancer [17]. A proposed mechanism of synergy is synthetic lethality leading to mitotic catastrophe [18].

Gemcitabine has been suggested to deplete ATP, which may augment alisertib binding to the ATP-site of AKA [19]. Additionally, AKA upregulates NF-κB whose expression can be suppressed with aurora kinase inhibition [20, 21]. This is especially important in pancreatic cancer which usually demonstrates NF-κB activation, and is consistent with downstream signaling activation from Kras mutations which are present in the majority of pancreatic cancers [22, 23]. To our knowledge, the combination of alisertib and gemcitabine has not yet been tested for treatment of pancreatic cancer.

## Materials and methods

This clinical trial (NCT01924260) was conducted following all applicable regulatory requirements and was approved by the UC Davis Institutional Review Board. All participating subjects provided written informed consent prior to initiation of trial-associated procedures and treatment.

### Study design and treatment

This was an open-label phase I clinical trial with two-phase design including a dose escalation and an expansion phase in pancreatic cancer. In the dose escalation phase, a standard 3 + 3 design was used to determine the maximum tolerated dose (MTD) of alisertib in combination with gemcitabine. Alisertib was administered orally twice daily (BID) on days 1–3, 8–10, and 15–17 of a 28-day treatment cycle. Gemcitabine was given concurrently at standard dosing of 1000 mg/m^2^ intravenously on days 1, 8, 15. A starting dose of alisertib 20 mg BID was used in the dose escalation phase and was escalated in cohorts of at least three evaluable subjects at 30 mg BID, 40 mg BID, and 50 mg BID until MTD or the highest feasible dose level (Table S1). The dose of 50 mg BID was the highest dose allowable and was previously reported as the recommended phase II dose (RP2D) for alisertib monotherapy [12, 24, 25]. In the expansion phase, subjects received the MTD or RP2D following the same cycle schedule. Dose adjustments were allowed for both drugs (Table S1B, 1C).

Treatment with alisertib and gemcitabine was repeated every 28 days. To proceed to the next cycle, lab parameters included ANC ≥ 1500/mm^3^ and platelet count ≥ 100,000/mm^3^, and all other toxicity considered by the investigator to be related to therapy with alisertib or gemcitabine must have resolved to grade ≤ 1 or to the subject’s baseline values. If the subject failed to meet the above-cited criteria for initiating a cycle, then the next treatment was delayed for up to 1 week. Thereafter, the subject was re-evaluated to determine continuation eligibility. Dose modification (Table S1B, 1C) was required for cycle initiations delayed > 1 week due to incomplete recovery from treatment-related toxicity.

### Objectives and statistical considerations

The primary objective of this phase I study was to determine the MTD and RP2D of alisertib in combination with gemcitabine. Upon determination of the MTD, an expansion cohort of subjects with pancreatic cancer was enrolled to further evaluate safety and evidence of clinical activity. Secondary objectives included preliminary efficacy as determined by objective response rate (ORR) and progression-free survival (PFS), and effects of alisertib drug–drug interactions on the pharmacokinetics of gemcitabine and its primary metabolite, 2′, 2′-difluorodeoxyuridine (dFdU).

The MTD was defined as the highest dose tested in which fewer than 33% of subjects experienced DLT attributable to the study drugs, when at least six subjects were treated at that dose and evaluable for toxicity. The RP2D was to be selected based on the totality of safety and efficacy data, and did not necessarily equal the MTD. If the MTD was not reached by dose level 4, then that dose level would be the RP2D.

After identification of the MTD or RP2D, goal accrual was 16 subjects at that dose (6 patients in dose escalation and 10 patients in dose expansion) which allowed for an 81% chance of seeing at least 1 example of any toxicity that occurs in 10% or more of similar subjects, and a 93% chance of seeing at least one example of any toxicity that occurs in 15% or more of similar subjects.

### Subject selection

Eligible subjects were required to be ≥ 18 years of age, have an Eastern Cooperative Oncology Group (ECOG) performance status of 0 to 2, and be able to swallow and retain oral medications. Female subjects of childbearing age were required to be willing to use effective birth control for the duration of the study. Male subjects were required to agree to use effective contraception during the entire study and for 4 months after the last dose of alisertib. Other eligibility criteria included: adequate bone marrow defined as absolute neutrophil count (ANC) ≥ 1500/mm^3^ and platelet count  ≥ 100,000/mm^3^, adequate hepatic function defined as total bilirubin with institutional normal limits and ALT and AST ≤ 2.5 times institutional upper limit of normal or  ≤ 5 times institutional upper limit of normal in presence of liver metastases, and adequate renal function defined as creatinine  ≤ 1.5 times institutional upper limit of normal or creatinine clearance  > 60 ml/min/1.73m^2^ measured by 24-h urine collection. Prior treatment with chemotherapy, immunotherapy, targeted therapy, or radiation must have been completed at least 2 weeks prior to start of protocol treatment and side effects related to prior treatment (excluding alopecia, lymphopenia, and hyperglycemia) resolved to grade ≤ 1. Prior gemcitabine-based regimens in the palliative setting were permitted if there was no evidence of progression on therapy or at least 6 months had elapsed after discontinuation of gemcitabine-based treatment. Prior gemcitabine in the adjuvant setting was permitted if the last treatment was greater than 6 months prior to registration.

In addition to the above criteria, criteria specific to subjects enrolled in the dose escalation phase of the trial included histologically or cytologically confirmed metastatic or unresectable solid tumor and any number of prior chemotherapies. Subjects enrolled in the dose expansion phase were required to have histologically or cytologically confirmed metastatic or unresectable pancreatic adenocarcinoma, up to 2 prior chemotherapy regimens in the palliative setting. Measurable disease was only required for the dose expansion phase.

Exclusion criteria for both cohorts included: prior treatment with AKA-targeted drugs, history of Gilbert’s syndrome (due to metabolism of alisertib via glucuronidation), significant history of cardiac disease, symptomatic or uncontrolled brain metastases, prior radiation to greater than 25% of bone marrow or whole pelvis radiation, anticoagulation with warfarin, active clinical infection including active HIV, chronic hepatitis B, and pregnant or breast-feeding female subjects.

All subjects provided written consent. The study was approved by the UC Davis institutional review board (IRB) and was compliant with Good Clinical Practices guidelines and the Declaration of Helsinki.

### Safety and efficacy assessments

Safety was monitored by performing physical examination and assessing vital signs, performance status, laboratory evaluations and an ECG as well as by collecting adverse events at every study visit. Toxicity was evaluated according to the National Cancer Institute Common Terminology Criteria for Adverse Events (NCI CTCAE, version 4.02). All subjects receiving any amount of study drug were evaluable for toxicity.

DLT was defined as any related (possibly, probably, or definitely) grade 3 non-hematologic toxicity or any attributable grade 4 toxicity. Grade 3 nausea or emesis was not considered dose-limiting unless it did not reverse to grade ≤ 2 within 96 h of appropriate management. Grade 3 fatigue was not considered dose-limiting unless it did not reverse to grade ≤ 2 in 7 days. Transient grade 4 neutropenia was not considered dose-limiting unless it did not resolve to grade 3 within 7 days or was associated with febrile neutropenia. DLT assessment was based on the first cycle of treatment. To be evaluable for DLT, a subject had to receive ≥ 80% of the total intended dose of both gemcitabine and alisertib and be observed for at least 3 weeks after the start of the first cycle or have experienced a DLT. All subjects enrolled were followed for DLT, and any subjects who were not evaluable for toxicity were replaced.

Response was assessed using CT scans of the chest, abdomen, and pelvis (MRI could be substituted for abdomen and pelvis) at baseline and every 2 cycles on study per the Response Evaluation Criteria in Solid Tumors guideline (RECIST, version 1.1). PFS was evaluated using Kaplan–Meier survival analysis.

Adverse events were summarized according to organ system, laboratory category, and dose level in frequency tables graded according to CTCAE v4.02. Information regarding each subject’s course including completion of therapy, dose delays, premature discontinuation, and major protocol violations were tabulated and summarized.

### Pharmacokinetic (PK) assessments

Pharmacokinetic sampling was performed on the expanded cohort of subjects with pancreatic cancer treated at the RP2D. Alisertib was not administered on cycle 1, day 1 to enable gemcitabine pharmacokinetic sampling on day 1 and day 2 of cycle 1 in the absence of co-administered alisertib. This served as the reference baseline for comparison to gemcitabine pharmacokinetics when co-administered with alisertib on day 8 of cycle 1. On cycle 1 day 8, alisertib was administered within 10 min of the start of the gemcitabine infusion, and the second (evening) dose of alisertib was not administered to allow for gemcitabine measurement at the 24 h timepoint. Both the parent drug (gemcitabine, dFdC) and the metabolite (dFdU) were measured to evaluate changes in gemcitabine metabolism related to alisertib administration. Pharmacokinetic blood samples were collected with dipotassium ethylenediaminetetraacetic acid (K2EDTA) as the anticoagulant before treatment, immediately after gemcitabine infusion, and at additional pre-specified post-infusion timepoints. Actual timepoints were recorded for PK parameter evaluation. Measurement of plasma gemcitabine, dFdU, and alisertib concentrations are described in the Supplementary Methods.

Gemcitabine, dFdU and alisertib PK parameters were estimated using non-compartmental analysis (NCA) with Certara Phoenix WinNonlin 8.0 (Princeton, NJ), including mean peak concentration (*C*_max_) and exposure (Area Under the Curve, AUC). The AUC parameters were estimated using the linear-up-log-down trapezoidal rule. Tests of significance between day 1 and day 8 PK parameters were performed using paired t-tests with GraphPad Prism 6.07 (San Diego, CA).

### AKA and pHH3 immunohistochemical staining assessments

When available, subject archival tumor specimens were collected for immunohistochemical analysis of AKA level and proliferative index assessment. Paraffin-embedded tumor sections were cut at 5 µm and immunostained for AKA (mouse clone JLM28, Leica Biosystems, Buffalo Grove, IL; manual detection using a mouse HRP polymer) and phosphohistone H3 (pHH3, rabbit polyclonal, Millipore Sigma, Burlington, MA; manual detection using a rabbit HRP polymer). pHH3 is a marker of mitotic activity and a more specific marker than Ki-67, which is expressed during all active phases of the cell cycle.

AKA and pHH3 stained slides were scored using the following criteria: each tumor was scanned for “hot spots” of AKA expression, defined as areas of tumor with the highest density of immunopositive tumor nuclei according to visual scanning at low-power magnification. Nuclear staining (with or without cytoplasmic staining) of at least 2 + (intermediate) intensity (range 0–3, as per Allred scoring method [26]) was considered positive. Each hot spot was photocaptured at 200× magnification at the same level of illumination and individual tumor nuclei were counted. Tumor nuclei with 1+ or less intensity nuclear staining were counted as negative. A minimum of 500 cells were counted for each tumor (range 500–2082). pHH3 expression was assessed in the exact same area that the AKA count was performed using the same criteria.

## Results

### Clinical characteristics

Between August 2013 and October 2016, a total of 26 subjects (median age 57 years, 13 men, 13 women) were enrolled at UC Davis Comprehensive Cancer Center. Subject characteristics are shown in Table 1. Twenty-one subjects were enrolled in the dose escalation phase with an additional 5 subjects enrolled and treated in the expansion phase. A total of 14 subjects were treated at dose level 4 (9 in dose escalation and 5 in dose expansion). Enrollment into dose expansion was discontinued due to poor accrual. Primary malignancies included non-small cell lung cancer (NSCLC), pancreatic cancer, and colorectal cancer, among others. A majority of subjects (77%) were treated with at least 2 prior lines of systemic therapy.

**Table 1:** Baseline demographic and clinical information for all subjects

Characteristic	Total	Dose escalation	Dose expansion
	(*N* = 26)	(*N* = 21)	(*N* = 5)
Age–median (range)	57 (42–82)	57 (42–75)	63 (48–82)
Sex			
Male	13	10	3
Female	13	11	2
ECOG performance status			
0	9	7	2
1	16	13	3
2	1	1	
Primary diagnosis			
NSCLC	7	7	
Colorectal	3	3	
Neuroendocrine (poorly differentiated)	3	3	
SCLC	2	2	
Head and neck	2	2	
Pancreas	6	1	5
Gallbladder	1	1	
Small bowel	1	1	
Mesothelioma	1	1	
Prior lines of chemo			
0–1	6	4	2
2–3	16	13	3
4 +	4	4	
Assigned dose level			
1	3	3	
2	3	3	
3	6	6	
4	14	9	5

### DLT and MTD

In the dose escalation phase, 21 subjects were evaluable for DLT. DLTs were observed in one out of 6 subjects treated at dose level 3 (grade 3 urinary tract infection; subject 7) and 1 out of 6 subjects treated at dose level 4 (grade 3 mucositis, lymphopenia, leukopenia, febrile neutropenia, hyponatremia, and dehydration; subject 19). Subject 7 was hospitalized on cycle 1 day 7 for septic shock from presumed urinary source requiring 3-day hospitalization for pressor support, fluid hydration, and antibiotics. Urinary tract infection was felt possibly related to both drugs. Subject 19 presented on cycle 1 day 8 with neutropenic fever, mucositis, hyponatremia, and dehydration. Despite antibiotics and resolution of neutropenia, fevers persisted and no infectious source was identified, although the subject was treated empirically for candida esophagitis. This subject was ultimately discharged on hospice. All grade 3 events were felt possibly or probably related to either study drugs. No DLTs were observed at dose level 1 or 2. The MTD and RP2D was determined to be dose level 4.

### Treatment exposures, delays, and dose reductions

**Fig. 1:**
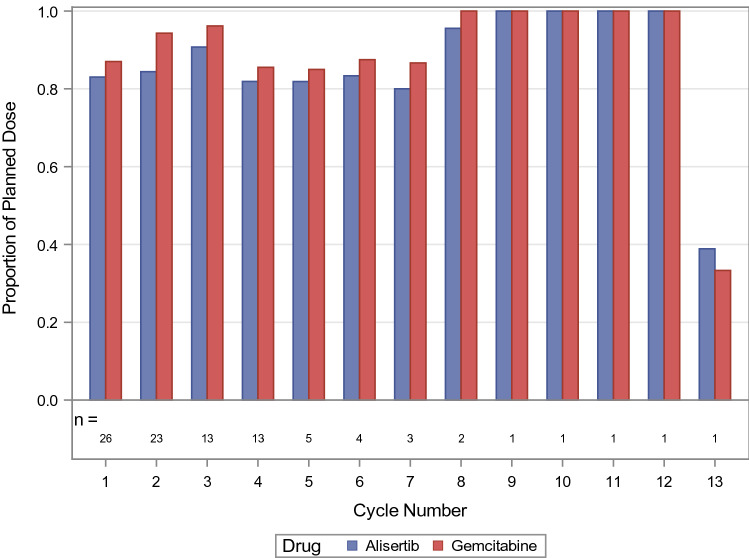
Proportion of planned dose delivered for alisertib (blue) and gemcitabine (red) in all treatment cycles for all subjects. For each cycle, the proportion of planned dose was calculated by the total dose delivered divided by total planned dose according to alisertib dose level and gemcitabine 1000 mg/m^2^

A total number of 94 cycles of alisertib and gemcitabine were delivered among the 26 subjects enrolled. Median number of cycles per subject was 3 (range 1–13), and median duration of treatment was 2.84 months (range 0.36–13.22). Similar median duration of treatment and cycles delivered were observed between the dose levels (Table S2). Proportion of planned dose received, defined as total dose received divided by total planned dose according to the dose level for alisertib and 1000 mg/m^2^ for gemcitabine, was calculated according to each subject (Table S3) and each cycle (Fig. 1).

For alisertib, 18 subjects underwent dose delays/omissions and 9 subjects underwent dose reductions. A total of 27 cycles of alisertib were modified. For gemcitabine, 16 subjects required dose delays, 14 subjects required dose omission, and 5 subjects required dose reductions. A total of 17 cycles had dose omissions, 10 cycles were given at a reduced dose, and 43 cycles had delayed doses. Although dose delays were common for gemcitabine, subjects still received the majority of the planned doses (Table S3, Fig. 1).

### Safety and toxicity

Treatment-related adverse events (Table 2) were observed in all subjects and included leukopenia (100%), neutropenia (88%), thrombocytopenia (88%), anemia (81%), lymphopenia (77%), fatigue (69%), mucositis (62%), and ALT elevation (62%). Grade ≥ 3 adverse events were observed in 92% of all subjects and predominantly hematologic, including neutropenia (65%), leukopenia (58%), lymphopenia (46%), and anemia (31%). Similar adverse events were seen at dose level 4, and all 14 subjects experienced grade ≥ 3 adverse events and neutropenia, of which 79% were grade ≥ 3 with one patient having febrile neutropenia..

**Table 2:** Treatment-related adverse events occurring in > 10% of subjects or serious adverse events

Event, *N* (%)	All subjects (*N* = 26)		Dose level 4 (*N* = 14)	
	Any grade	Grade ≥ 3	Any grade	Grade ≥ 3
Any	26 (100)	24 (92)	14 (100)	14 (100)
Hematologic				
Anemia	21 (81)	8 (31)	12 (86)	2 (14)
Leukopenia	26 (100)	15 (58)	14 (100)	10 (71)
Lymphopenia	20 (77)	12 (46)	10 (71)	6 (43)
Lymphocytosis	2 (8)	2 (8)	2 (14)	2 (14)
Neutropenia	23 (88)	17 (65)	14 (100)	11 (79)
Febrile neutropenia	1 (4)	1 (4)	1 (7)	1 (7)
Thrombocytopenia	23 (88)	4 (15)	13 (93)	2 (14)
Constitutional				
Fatigue	18 (69)	1 (4)	11 (79)	1 (7)
Myalgia	2 (8)	1 (4)	1 (7)	1 (7)
Metabolic				
Total bilirubin elevated	3 (12)	0	1 (7)	0
AST elevated	15 (58)	1 (4)	7 (50)	1 (7)
ALT elevated	16 (62)	1 (4)	7 (50)	1 (7)
ALP elevated	11 (42)	1 (4)	6 (43)	1 (7)
Creatinine elevated	6 (23)	1 (4)	1 (7)	1 (7)
Albumin decreased	11 (42)	1 (4)	5 (36)	0
Hypoglycemia	4 (15)	0	2 (14)	0
Potassium decreased	8 (31)	1 (4)	5 (36)	1 (7)
Magnesium decreased	3 (12)	0	1 (7)	0
Sodium decreased	11 (42)	5 (19)	6 (43)	3 (21)
Phosphate decreased	5 (19)	2 (8)	3 (21)	1 (7)
Gastrointestinal				
Mucositis oral	16 (62)	6 (23)	11 (79)	3 (21)
Oral pain	3 (12)	0	2 (14)	0
Nausea	14 (54)	1 (4)	10 (71)	1 (7)
Vomiting	10 (38)	1 (4)	5 (36)	0
Diarrhea	10 (58)	3 (12)	8 (57)	3 (21)
Constipation	6 (23)	0	0	0
Anorexia	5 (19)	0	4 (29)	0
Dry mouth	2 (8)	0	2 (14)	0
Dehydration	2 (8)	2 (8)	2 (14)	2 (14)
Esophagitis	1 (4)	1 (4)	1 (7)	1 (7)
Dermatologic				
Alopecia	3 (12)	0	2 (14)	0
Pruritus	3 (12)	0	2 (14)	0
Neurologic				
Dizziness	3 (12)	0	2 (14)	0
Headache	3 (12)	0	2 (14)	0
Lymphatic				
Edema, limbs	3 (12)	0	0	0
Cardiac				
Pericardial effusion	1 (4)	1 (4)	0	0
Respiratory				
Dyspnea	3 (12)	1 (4)	1 (4)	0
Pleural effusion	1 (4)	0	0	0
Infection				
Urinary tract infection	1 (4)	1 (4)	0	0

### Serious adverse events

Serious treatment-related adverse events were observed in 6 subjects (23%). Subject 2 had pericardial effusion (grade 4), pleural effusion (grade 2), and dyspnea (grade 3). Pericardial effusion occured during cycle 14, was not malignant, and felt possibly related to study drugs; however, this resulted in cardiac tamponade requiring a pericardial window. Subject 7 had a urinary tract infection (grade 3), which was classified as a DLT. Subject 22 had hyponatremia (grade 3), mucositis (grade 3), and hematologic abnormalities (grade 4 leukopenia, neutropenia, lymphopenia; grade 3 anemia). Subject 14 had anemia (grade 3), and subject 19 had febrile neutropenia (grade 3) and mucositis (grade 3). Subject 23 had dehydration (grade 3), diarrhea (grade 3), acute kidney injury (grade 3), and vomiting (grade 1).

### Response

**Fig. 2:**
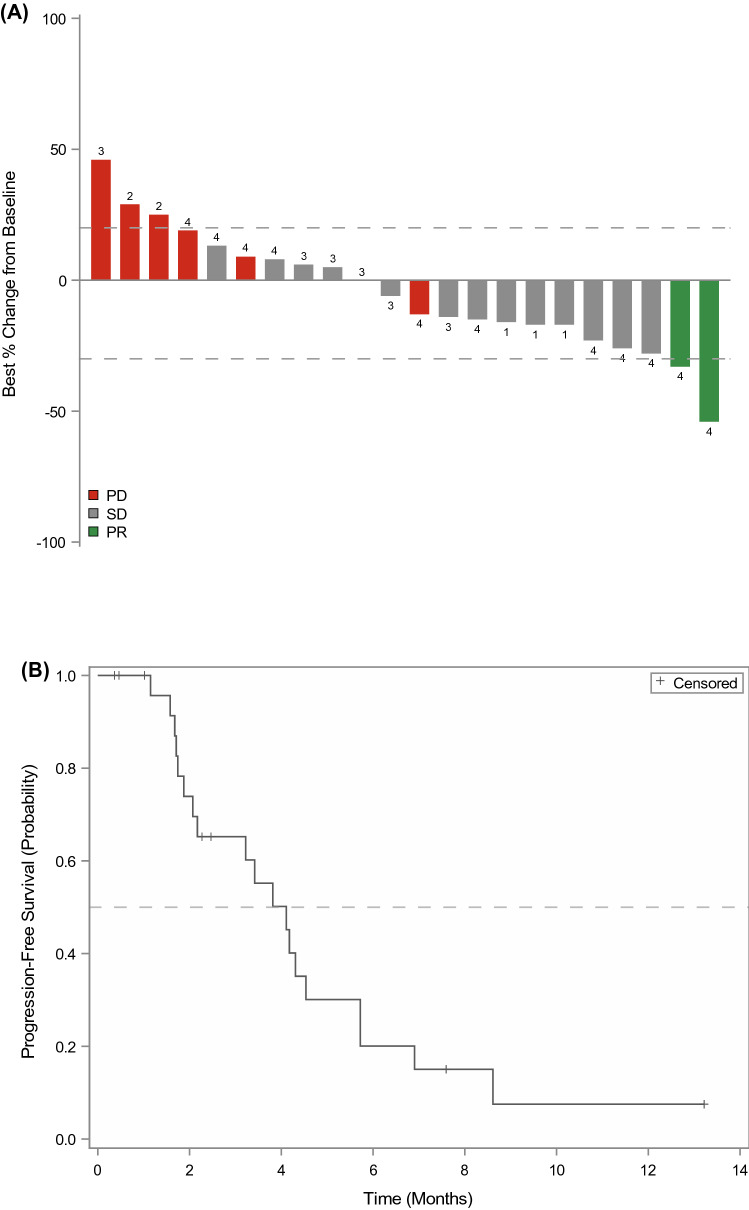
**A** Waterfall plot of best responses per evaluable subject. Dashed lines represent +20% and −30% change from baseline. 1–4: corresponding dose levels, PD: progressive disease, SD: stable disease, PR: partial response. **B** Kaplan–Meier plot for progression-free survival. Median PFS was 4.1 months (95% CI 2.1–4.5)

Response was evaluable in 22 subjects. Best response of partial response, stable disease, and progressive disease was observed in 2 (9%), 14 (64%), and 6 (27%) subjects, respectively (Fig. 2A). Responses were observed in one subject with lung adenocarcinoma and one subject with pancreatic adenocarcinoma. Median PFS was 4.1 months (95% CI 2.1–4.5, Fig. 2B).

Eighteen subjects had archival tumor specimens evaluable for immunohistochemical evaluation of AKA and pHH3 and radiographic response (Table S4). Staining for AKA and pHH3 was generally low (AKA average 5.9% positive tumor nuclei, range 0–22.4%; pHH3 average 7.8% positive tumor nuclei, range 0–98%), although one subject was remarkable for pHH3 staining of 98.0% of evaluated tumor cells with best response of stable disease. Disease control and partial responses were observed regardless of AKA and pHH3 expression. PFS did not appear to correlate to AKA or pHH3 expression.

### Pharmacokinetics

**Fig. 3:**
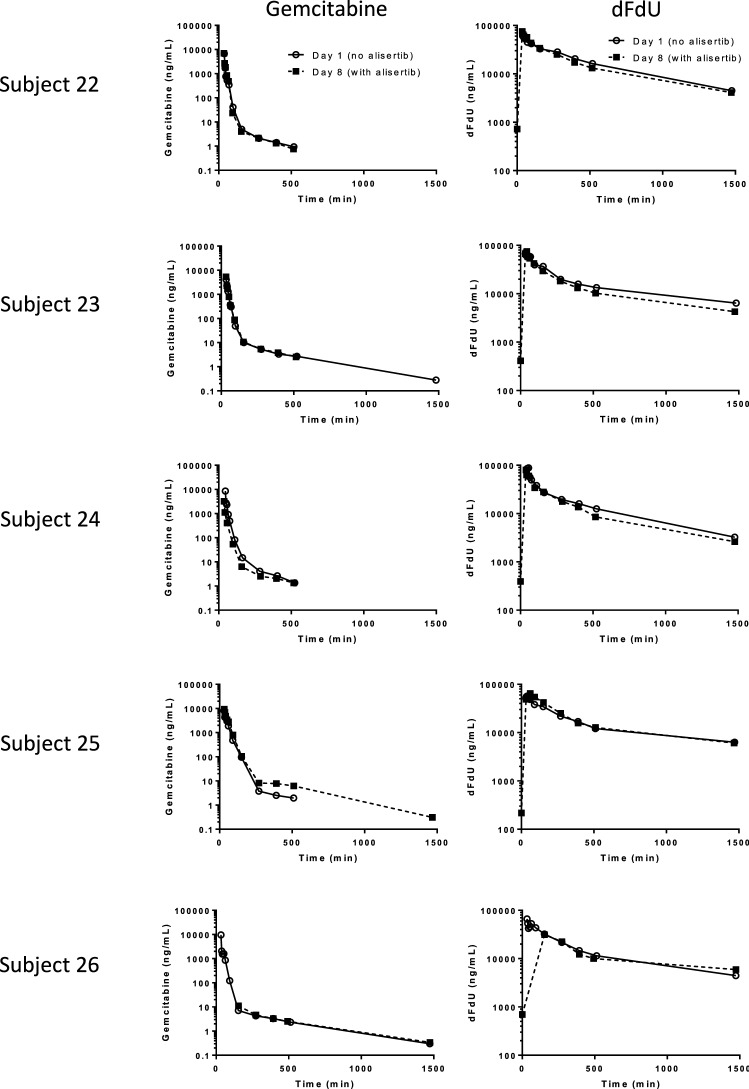
Gemcitabine (left panels) and dFdU (right panels) concentration–time profiles for subjects (22–26) evaluable for gemcitabine and dFdU PK on day 1 (no alisertib, open markers) and on day 8 (with alisertib, closed markers in black)

The PK parameters including *C*_max_, AUC_0->last_, AUC_0->inf_, and the percentage of extrapolated AUC_0->inf_ of both gemcitabine and dFdU (Table 3) on day 1 (without alisertib) and day 8 (with alisertib) were evaluated by NCA with WinNonlin. One subject missed the PK day 8 timepoint and therefore was excluded from the gemcitabine NCA. The NCA PK parameters of alisertib were also evaluated (Table S5). Another subject had *C*_max_ at the last timepoint and therefore was excluded from the alisertib NCA since both the actual *C*_max_ and AUC_0->inf_ were unable to be estimated. All NCA PK parameters for gemcitabine and dFdU were not significantly affected by co-administered alisertib among individual subjects (paired *t* test, *P* > 0.05). The gemcitabine and dFdU concentration–time profiles on day 1 (no alisertib) and day 8 (with alisertib) within each subject were also visually overlapping (Fig. 3), suggesting the low possibility of DDI effect from alisertib on gemcitabine. Alisertib PK profiles on day 8 appeared similar between subjects (Fig. S1). Subject 22 (Fig. S1) had an unexpectedly high C_max_ (1750 ng/mL), and Subject 24 (Fig. S1) had an unexpectedly long *T*_max_ (24 h) when compared to other subjects here (1.5–4.5 h) and in published literature (3-4 h) [30]. No offending concomitant medications were noted to affect the PK parameters of these two subjects, and no aberrancies were noted in drug administration dosage or timing.

**Table 3:** Estimated PK parameters of non-compartment analysis for gemcitabine. Day 1 (without alisertib) and Day 8 (with alisertib) were compared using paired t testing and no significant differences were found (*p* > 0.05) for (a) gemcitabine and (b) dFdU

Parameters	Unit	Day 1			Day 8		
		*N*	Mean	SD	*N*	Mean	SD
Gemcitabine							
C_max_	mcg/mL	5	7.42	2.13	4	6.24	2.62
AUC_0->last_	min*mcg/mL	5	215.88	68.00	4	193.81	117.43
AUC_0->inf_	min*mcg/mL	5	216.19	68.15	4	194.21	117.20
Percentage extrapolated AUC_0->inf_	%	5	0.14	0.07	4	0.32	0.30
dFdU							
*C*_max_	mcg/mL	5	67.92	12.13	4	73.75	6.48
AUC_0->last_	min*mcg/mL	5	2.18 × 10^4^	1.60 × 10^3^	4	2.06 × 10^4^	2.92 × 10^3^
AUC_0->inf_	min*mcg/mL	5	2.47 × 10^4^	4.93 × 10^3^	4	2.18 × 10^4^	1.60 × 10^3^
Percentage extrapolated AUC_0->inf_	%	5	18.13	7.49	4	15.73	6.17

## Discussion

An alternative approach to chemotherapeutic, specifically taxane-induced mitotic inhibition, is to specifically target proteins critical to mitosis and only expressed in cells at the time of mitosis. Thus, there has been increasing interest in developing drugs to target proteins such as AKA that meet this description. The addition of gemcitabine may enhance alisertib activity by capitalizing on DNA instability. We therefore sought to evaluate the safety and preliminary efficacy of alisertib in combination with gemcitabine in this phase I dose escalation and expansion trial.

Alisertib with gemcitabine proved to be a feasible drug combination, and a majority of subjects were able to be treated at the highest dose level (DL4) and RP2D of gemcitabine (1000 mg/m^2^ days 1, 8, 15) and alisertib 50 mg twice daily (days 1–3, 8–10, 15–17) in 28-day cycles. However, toxicity including hematologic abnormalities, fatigue, transaminitis, and mucositis resulted in frequent dose interruptions and reductions. Although subjects enrolled in this study were heavily pre-treated, disease control was noted in 16 evaluable subjects including two subjects with partial response. Although no clear relationship between efficacy and AKA or pHH3 expression was observed in these data, exploratory biomarkers in small cell lung cancer have demonstrated a potential benefit signal in certain genomic subsets, namely alterations in cell cycle regulation genes [17]*.*

The metabolism of alisertib and its potential of perpetrating drug–drug interactions (DDIs) to other co-administered drugs has been extensively studied [27]. Briefly, the two main metabolites of alisertib are o-desmethyl alisertib and alisertib acyl glucuronide. In vitro phenotyping has demonstrated that CYP3A is largely involved in oxidative metabolism of alisertib. The acyl glucuronidation of alisertib is mainly due to uridine 5′-diphospho-glucuronosyltransferase (UGT) isoenzymes. In contrast, gemcitabine is primarily metabolized to dFdU in the liver and plasma by cytidine deaminase (CDA) for further clearance [28]. The CDA and transporter gene polymorphisms are responsible for the variance of metabolism and response [29]. In this study, we observed that gemcitabine PK parameters were not significantly affected by alisertib co-administration. In vitro, alisertib and its 2 main metabolites have not shown appreciable inhibition to selected CYPs [27], thus implying a low likelihood of perpetrating DDIs with co-administrated drugs through CYP and UGT inhibition/activation. Alisertib was orally administrated within 10 min after the starting of gemcitabine infusion. Although alisertib PK parameters were not compared between the presence and absence of gemcitabine intrasubject, similar *C*_max_ (700–900 ng/mL) and AUCs for alisertib were observed here as has been previously reported [30]. This is the first clinical DDI study for alisertib and gemcitabine. It is not yet known whether gemcitabine exerts DDI to alisertib. Further evaluation is required to confirm alisertib’s effects on CDA-mediated gemcitabine metabolism in vitro. Our current finding suggests low possibility of DDI between the two drugs.

The RP2D of alisertib monotherapy has previously been reported as 50 mg twice daily for 7 days in a 21-day cycle [12, 24, 25]. In combination with paclitaxel, RP2D has been 40 mg twice daily at a schedule similar to the one reported here [31]. Tolerability issues have been raised with alisertib in either monotherapy or combination therapy. In a monotherapy trial of solid tumors, treatment-related adverse events included neutropenia (43%), leukopenia (21%), and anemia (10%), and a high rate of SAEs (43%) [32]. In combination with paclitaxel, tolerability concerns have included high rates of neutropenia, grade ≥ 3 events, SAEs, and dose reductions or discontinuations [17, 31], although health-related quality of life appeared similar to paclitaxel alone [31]. Similarly, diarrhea, dehydration, and hematologic toxicities have been dose-limiting for alisertib plus irinotecan [33].

Alisertib monotherapy among multiple solid organ malignancies has demonstrated limited efficacy with response rates of approximately 5–20% depending on the primary tumor subtype [32]. Combination alisertib and paclitaxel has been evaluated in randomized phase 2 trials of subjects with small cell lung cancer and breast/ovarian cancer. In small cell lung cancer, alisertib and paclitaxel has been compared to placebo and paclitaxel with 89 subjects in each arm. Although the primary endpoint of PFS was not met, the alisertib and paclitaxel arm trended toward improved PFS (mPFS 3.32 vs 2.17 months; HR 0.77, 95% CI 0.557–1.067, *p* = 0.113), and response rates were comparable (22 vs 18%) [17]. Similar findings have been noted in breast/ovarian subjects (mPFS 6.7 vs 4.7 months; HR 0.75, 80% CI 0.58–0.96, *p* = 0.14) with similar response rates in the two arms (60% vs 52%, *p* = 0.38) [31]. In acute myelogenous leukemia (AML), induction 7 + 3 chemotherapy plus alisertib 30 mg twice daily on days 8–15 has shown preliminary efficacy with complete remission rates of 64%. This is notable especially since subjects were required to have high-risk features [34].

Alisertib plus gemcitabine has a rational basis for combination in advanced solid organ malignancies. Alisertib co-administration did not affect gemcitabine PK characteristics, suggesting against any DDIs of alisertib on gemcitabine. Preliminary efficacy in terms of disease control among a heavily pre-treated group of subjects was observed, and toxicity, predominantly gastrointestinal and hematologic, was manageable. Further evaluation of this combinatorial strategy would benefit from the identification of a predictive biomarker(s).

## Supplementary Information

Below is the link to the electronic supplementary material.

Supplementary file1 (DOCX 16 KB): **Table S1** A) Dose Escalation Schema, B) MLN8237 Dose Adjustments, C) Gemcitabine Dose Adjustments

Supplementary file2 (DOCX 15 KB): **Table S2** Median duration on treatment and number of cycles per dose level

Supplementary file3 (DOCX 19 KB): **Table S3** Number of cycles, dose level, alisertib dose, proportion of total planned dose received for alisertib and gemcitabine, and treatment duration per subject

Supplementary file4 (DOCX 17 KB): **Table S4** AKA IHC score, pHH3 IHC score, best response, and PFS among subjects evaluable for IHC and response. A minimum 500 total cells was required for IHC evaluation. PD: progressive disease, PFS: progression-free survival, PR: partial response; SD: stable disease, *: censored

Supplementary file5 (DOCX 15 KB): Estimated PK parameters of non-compartment analysis for alisertib

Supplementary file6 (PDF 52 KB): **Fig.** S**1** Alisertib plasma concentration-time profile for subjects (22-26) evaluable for alisertib PK

Supplementary file7 (DOCX 16 KB): Supplementary Methods

## Data Availability

The datasets generated during and/or analyzed during the current study are available from the corresponding author on reasonable request.
