# Supplementary material for: A phase I dose escalation, dose expansion and pharmacokinetic trial of gemcitabine and alisertib in advanced solid tumors and pancreatic cancer
Source: Cancer Chemother Pharmacol. 2022 Jul 30;90(3):217–28. doi: 10.1007/s00280-022-04457-9 (PMC9402746; doi:10.1007/s00280-022-04457-9)
Supplement: Supplementary file 1 — Supplementary file1 (DOCX 16 KB): Table S1 A) Dose Escalation Schema, B) MLN8237 Dose Adjustments, C) Gemcitabine Dose Adjustments [file 280_2022_4457_MOESM1_ESM.docx]

A)

| **Dose Level** | **Gemcitabine**  **(mg/m^2^ IV)**  **days 1, 8, 15 every 28 days** | **MLN8237**  **(mg PO BID)**  **1-3, 8-10, 15-17days** |
| --- | --- | --- |
| 1 | 1000 | 20 |
| 2 | 1000 | 30 |
| 3 | 1000 | 40 |
| 4 | 1000 | 50 |

| B) | | |
| --- | --- | --- |
| **MLN8237 Level** | **Dose** | **Schedule** |
| 0 | Starting Dose | days 1-3, 8-10, 15-17 |
| -1 | Starting Dose -10 mg BID | days 1-3, 8-10, 15-17 |
| -2 | Starting Dose -20 mg BID | days 1-3, 8-10, 15-17 |
| -3 | Discontinue |  |
| Level 0 is the starting dose of MLN8237 for the assigned Dose Level | | |
| C) | | |
| **Gemcitabine Level** | **Dose** | **Schedule** |
| 0 | 1000 mg/m^2^ | IV day 1, day 8, day 15 |
| -1 | 750 mg/m^2^ | IV day 1, day 8, day 15 |
| -2 | 600 mg/m^2^ | IV day 1, day 8, day 15 |
| -3 | Discontinue |  |
|  | | |
