# Supplementary material for: A phase I dose escalation, dose expansion and pharmacokinetic trial of gemcitabine and alisertib in advanced solid tumors and pancreatic cancer
Source: Cancer Chemother Pharmacol. 2022 Jul 30;90(3):217–28. doi: 10.1007/s00280-022-04457-9 (PMC9402746; doi:10.1007/s00280-022-04457-9)
Supplement: Supplementary file 2 — Supplementary file2 (DOCX 15 KB): Table S2 Median duration on treatment and number of cycles per dose level [file 280_2022_4457_MOESM2_ESM.docx]

|  | Dose Level | | | |
| --- | --- | --- | --- | --- |
|  | **1**  **(n=3)** | **2**  **(n=3)** | **3**  **(n=6)** | **4**  **(n=14)** |
| Median Duration (months) | 3.2 | 2.3 | 3.3 | 2.9 |
| Median Cycles | 4 | 2 | 3 | 3 |
