# Supplementary material for: A phase I dose escalation, dose expansion and pharmacokinetic trial of gemcitabine and alisertib in advanced solid tumors and pancreatic cancer
Source: Cancer Chemother Pharmacol. 2022 Jul 30;90(3):217–28. doi: 10.1007/s00280-022-04457-9 (PMC9402746; doi:10.1007/s00280-022-04457-9)
Supplement: Supplementary file 3 — Supplementary file3 (DOCX 19 KB): Table S3 Number of cycles, dose level, alisertib dose, proportion of total planned dose received for alisertib and gemcitabine, and treatment duration per subject [file 280_2022_4457_MOESM3_ESM.docx]

| Subject ID | Dose Level | Assigned Alisertib Dose  (mg BID) | Cycles | Proportion Alisertib Dosed | Proportion Gemcitabine Dosed | Treatment Duration (months) |
| --- | --- | --- | --- | --- | --- | --- |
| 1 | 1 | 20 | 2 | 1.00 | 1.00 | 1.7 |
| 2 | 1 | 20 | 13 | 0.94 | 0.92 | 13.2 |
| 3 | 1 | 20 | 4 | 0.94 | 0.92 | 3.2 |
| 4 | 2 | 30 | 8 | 0.96 | 1.00 | 8.8 |
| 5 | 2 | 30 | 2 | 1.00 | 1.00 | 2.3 |
| 6 | 2 | 30 | 2 | 1.00 | 1.00 | 1.9 |
| 7 | 3 | 40 | 2 | 0.29 | 0.29 | 1.0 |
| 8 | 3 | 40 | 2 | 1.00 | 1.00 | 2.5 |
| 9 | 3 | 40 | 6 | 1.00 | 1.00 | 6.9 |
| 10 | 3 | 40 | 4 | 1.00 | 1.00 | 4.1 |
| 11 | 3 | 40 | 2 | 0.75 | 0.83 | 2.0 |
| 12 | 3 | 40 | 4 | 0.90 | 0.92 | 4.4 |
| 13 | 4 | 50 | 4 | 0.88 | 0.92 | 4.6 |
| 14 | 4 | 50 | 4 | 0.93 | 0.92 | 3.8 |
| 15 | 4 | 50 | 1 | 0.33 | 0.33 | 0.9 |
| 16 | 4 | 50 | 4 | 1.00 | 1.00 | 3.5 |
| 17 | 4 | 50 | 5 | 0.85 | 0.88 | 6.2 |
| 18 | 4 | 50 | 2 | 0.94 | 1.00 | 2.4 |
| 19 | 4 | 50 | 1 | 0.11 | 0.33 | 0.4 |
| 20 | 4 | 50 | 1 | 0.67 | 0.33 | 0.5 |
| 21 | 4 | 50 | 4 | 0.92 | 0.92 | 4.1 |
| 22 | 4 | 50 | 2 | 0.69 | 0.75 | 2.3 |
| 23 | 4 | 50 | 2 | 0.54 | 0.67 | 1.8 |
| 24 | 4 | 50 | 7 | 0.73 | 0.73 | 7.6 |
| 25 | 4 | 50 | 4 | 0.63 | 0.88 | 5.7 |
| 26 | 4 | 50 | 2 | 0.88 | 0.83 | 2.0 |
