# Supplementary material for: A phase I dose escalation, dose expansion and pharmacokinetic trial of gemcitabine and alisertib in advanced solid tumors and pancreatic cancer
Source: Cancer Chemother Pharmacol. 2022 Jul 30;90(3):217–28. doi: 10.1007/s00280-022-04457-9 (PMC9402746; doi:10.1007/s00280-022-04457-9)
Supplement: Supplementary file 4 — Supplementary file4 (DOCX 17 KB): Table S4 AKA IHC score, pHH3 IHC score, best response, and PFS among subjects evaluable for IHC and response. A minimum 500 total cells was required for IHC evaluation. PD: progressive disease, PFS: progression-free survival, PR: partial response; SD: stable disease, *: censored [file 280_2022_4457_MOESM4_ESM.docx]

| Subject ID | AKA (%) | pHH3 (%) | Best Response | PFS (months) |
| --- | --- | --- | --- | --- |
| 1 | 0.0 | 0.2 | SD | 1.6 |
| 2 | 0.0 | 0.0 | SD | 13.2^*^ |
| 5 | 22.4 | 8.8 | PD | 2.1 |
| 6 | 4.9 | 3.6 | PD | 1.7 |
| 7 | 18.0 | 98.0 | SD | 1.0^*^ |
| 8 | 7.6 | 0.7 | SD | 2.5^*^ |
| 9 | 0.0 | 1.0 | SD | 6.9 |
| 11 | 11.6 | 2.8 | PD | 1.9 |
| 12 | 6.8 | 4.5 | SD | 4.2 |
| 13 | 0.0 | 5.0 | SD | 4.5 |
| 17 | 0.2 | 0.0 | PR | 5.7 |
| 18 | 5.6 | 7.6 | PD | 2.2 |
| 21 | 9.5 | 5.2 | SD | 4.3 |
| 22 | 1.2 | 0.3 | SD | 2.3* |
| 23 | 0.0 | 0.0 | PD | 1.7 |
| 24 | 0.0 | 1.1 | PR | 7.6^*^ |
| 25 | 7.1 | 0.3 | SD | 5.7 |
| 26 | 12.0 | 1.7 | PD | 1.7 |
