# Supplementary material for: A phase I dose escalation, dose expansion and pharmacokinetic trial of gemcitabine and alisertib in advanced solid tumors and pancreatic cancer
Source: Cancer Chemother Pharmacol. 2022 Jul 30;90(3):217–28. doi: 10.1007/s00280-022-04457-9 (PMC9402746; doi:10.1007/s00280-022-04457-9)
Supplement: Supplementary file 5 — Supplementary file5 (DOCX 15 KB): Estimated PK parameters of non-compartment analysis for alisertib [file 280_2022_4457_MOESM5_ESM.docx]

| Parameters | Unit | N | Mean | SD |
| --- | --- | --- | --- | --- |
| C_max_ | ng/mL | 5 | 973.00 | 442.60 |
| T_max_ | min | 5 | 451.00 | 578.20 |
| AUC_0->last_ | min*ng/mL | 5 | 7.09×10^5^ | 2.86×10^5^ |
| AUC_0->inf_ | min*ng/mL | 4 | 9.77×10^5^ | 3.18×10^5^ |
| Percentage Extrapolated AUC_0->inf_ | % | 4 | 25.08 | 13.53 |
