# Supplementary figures and images for: A phase I dose escalation, dose expansion and pharmacokinetic trial of gemcitabine and alisertib in advanced solid tumors and pancreatic cancer
Source: Cancer Chemother Pharmacol. 2022 Jul 30;90(3):217–28. doi: 10.1007/s00280-022-04457-9 (PMC9402746; doi:10.1007/s00280-022-04457-9)

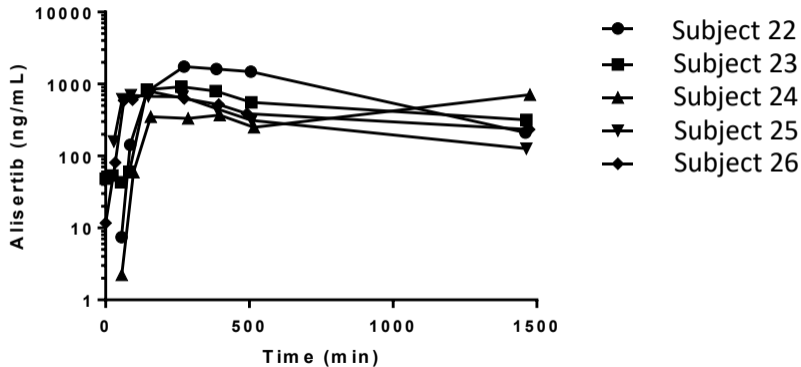

Supplement: Supplementary file 6 — Supplementary file6 (PDF 52 KB): Fig. S1 Alisertib plasma concentration-time profile for subjects (22-26) evaluable for alisertib PK [file 280_2022_4457_MOESM6_ESM.pdf]
