# Supplementary material for: A phase I dose escalation, dose expansion and pharmacokinetic trial of gemcitabine and alisertib in advanced solid tumors and pancreatic cancer
Source: Cancer Chemother Pharmacol. 2022 Jul 30;90(3):217–28. doi: 10.1007/s00280-022-04457-9 (PMC9402746; doi:10.1007/s00280-022-04457-9)
Supplement: Supplementary file 7 — Supplementary file7 (DOCX 16 KB): Supplementary Methods [file 280_2022_4457_MOESM7_ESM.docx]

Supplementary Methods:

Plasma gemcitabine and dFdU concentrations were determined by using a liquid chromatography tandem mass spectrometry (LC-MS/MS) bioanalytical methods on a Waters Xevo TQ-S UPLC-MS/MS system (Milford, MA). Multiple reaction monitoring was utilized to monitor the analytes in positive ionization mode, m/z 264.1→112.2 for gemcitabine, 265.1→113.2 for dFdU, and 269.1→153.1 for 5FdU (internal standard). Gemcitabine, dFdU and 5FdU were eluted at 0.47, 0.74 and 0.56 min, respectively. The linear quantitation ranges for both gemcitabine and dFdU were 0.25 to 250 ng/mL. The extraction yields were 70-100% for all analytes, and the matrix effects were less than 20%. Both inter- and intra-batch accuracy for quantifying both compounds (0.25, 0.75, 7.5, 75 and 156.25 ng/mL) were lower than 12% (deviation) except that gemcitabine had 19% of inter-batch deviation at 0.25 ng/mL. Both intra- and inter-batch precision were lower than 15%, except that dFdU was 16% at 0.25 ng/mL.

Plasma alisertib concentrations were quantitated by using an accurate LC-MS/MS method on an AB Sciex 4000 QTRAP tandem mass spectrometry system (AB Sciex, Framingham, MA) equipped with a Shimadzu Prominence Ultra-Fast Liquid Chromatography system (Shimadzu Corporation, Kyoto, Japan), which was validated in accordance with the FDA guidance^1^. Analytes were detected in positive ionization mode with multiple reaction monitoring, m/z 519.1→328.2 for alisertib and 477.2→316.1 for MLN8054 (internal standard), respectively. The linear calibration range was 3-1,500 ng/mL for alisertib. The extraction efficiency was over 90%, and the matrix effects were less than 13%. Accuracy (3, 9, 100 and 1000 ng/mL) ranged from 90.2% to 117%, and precision was < 10%. Deviation of 10-fold dilution is within 10%.

References:

1. U.S. Food and Drug Administration: Bioanalytical Method Validation. Guidance for Industry, 2018
